# Supplementary material for: Genomic insights into the virulence repertoire and hemibiotrophic lifestyle of the grapevine black rot pathogen Phyllosticta ampelicida
Source: G3 (Bethesda). 2025 Aug 19;15(10):jkaf186. doi: 10.1093/g3journal/jkaf186 (PMC12506662; doi:10.1093/g3journal/jkaf186)
Supplement: jkaf186_Supplementary_Data [file jkaf186_supplementary_data.zip › Supplementary_Figures_G3-2025-406089.pdf]

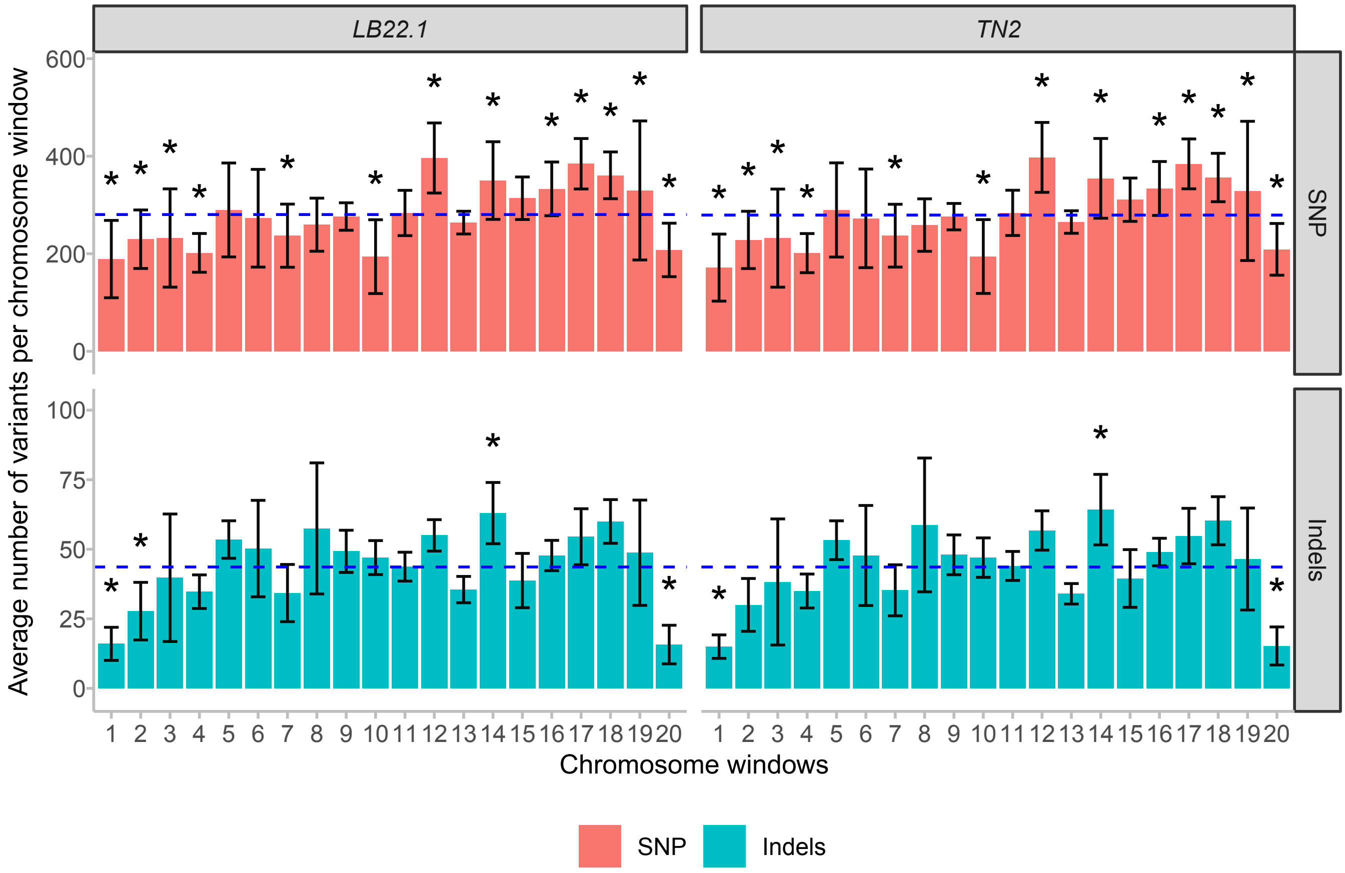

Supplementary Figure 1. Average number of variants per chromosome window. A chi-squared test was used to determine if the observed frequencies differ from expected frequencies. Significant p-values < 0.05 are represented by (*). Horizontal blue lines represets the expected frequency.

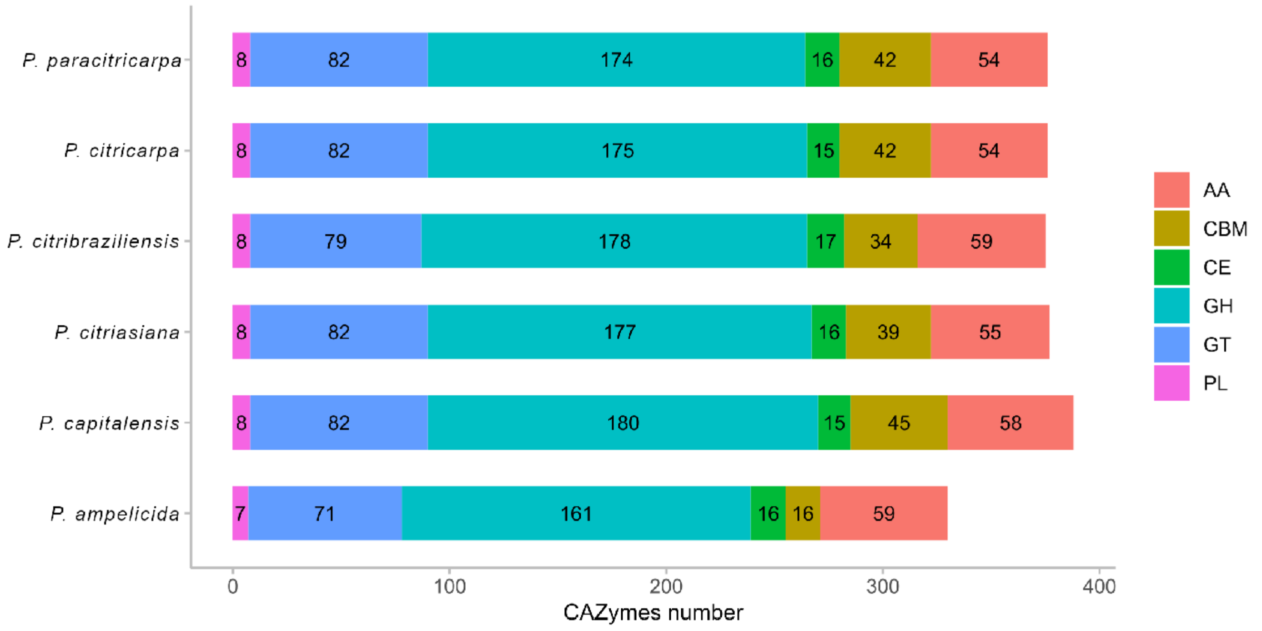

Supplementary Figure 2. Number of protein-coding genes annotated as CAZymes, divided in families, in Phyl-losticta species, as reported in this work and Bujis et al. (2021). AA: auxiliary activities; CBM: Carbohydrate-binding modules; CE: carbohydrate esterases; GH: glycoside hydrolases; GT: glyco-syltransferases; PL: polysaccharide lyases.
